# The effect of housing tenure on health status of migrant populations in China: are health service utilization and social integration mediating factors?

**DOI:** 10.1186/s13690-023-01218-9

**Published:** 2023-11-20

**Authors:** Fulin Jia, Xiaonan Liu, Yuxiang Wang, Mingze Ma

**Affiliations:** 1 Business School of Zhengzhou University, No.100, Science Avenue, Gaoxin District, Zhengzhou, 450001 Henan China; 2 School of Management, Zhengzhou University, No.100, Science Avenue, Gaoxin District, Zhengzhou, 450001 Henan China; 3 Department of Health Management, College of Public Health, Zhengzhou University, No.100, Science Avenue, Gaoxin District, Zhengzhou, 450001 Henan China

**Keywords:** Self-rated health, Housing tenure, Health service utilization, Social integration, Migrant populations

## Abstract

**Background:**

Current research suggests that there is an association between housing tenure and the health status of migrant populations, but the potential mediators of this association remain to be explored. We aimed to examine the effect of housing tenure on migrant populations’ health in China and how this effect is mediated by health service utilization and social integration.

**Methods:**

Data from the 2017 China Migrants Dynamic Survey of 47,459 participants was used. Logistic regression models were used to explore the effects of housing tenure, health service utilization, and social integration on the health status of migrant populations. Mediated effects models were used to explore the association among them. This study used the bootstrap method and KHB method to test the mediating effect of health service utilization and social integration.

**Results:**

Compared to private renters, owners with mortgages (OR: 0.828, 95% CI: 0.765–0.896) were significantly associated with a higher risk of poor health. Compared with private renters, outright owners were associated with a lower risk of poor health (OR: 1.016, 95% CI: 0.935, 1.104), but the association was not statistically significant (*p* > 0.05). Moreover, health service utilization (OR: 1.422, 95% CI: 1.268, 1.594) and social integration (OR: 4.357, 95% CI: 3.555, 5.341) were both significantly associated with a higher probability of good health (*p* < 0.001).

**Conclusion:**

Among migrant populations, homeowners with mortgages had a lower likelihood of good health than private renters, while there was no significant difference in the health status between outright owners and private renters. Moreover, health service utilization and social integration mediate the effect of housing tenure on the health status of migrant populations. Policies and interventions can be designed to improve the health service utilization and social inclusion of migrant populations to reduce health disparities across housing tenure types.

**Table:** 

**Text box 1. Contributions to the literature**
• Housing is a social determinant of health, and previous studies have shown that housing tenure has an impact on individuals’ health. Yet little research has focused on the pathways between housing tenure and the health status of migrant populations.
• The results show that housing tenure can partially affect health status of migrant populations through health service utilization and social integration.
• These findings contribute to explaining the main pathways of the impact of housing tenure on health and improving China’s migrant populations’ health status.

## Background

In recent years, China has experienced rapid urbanization, which has been accompanied by a massive movement of the population [1]. According to the latest statistics, the internal migrant population has increased to more than 375 million in 2020, representing more than 26% of the Chinese total population [2]. These migrants are one of the main productive forces in cities and make an important contribution to economic and social development [3]. Research on the health status of the migrant population is essential for understanding the actual situation of this population, improving their health, and promoting sustainable urban development.

Housing has long been recognized as a social determinant of health, and the relationship between housing and health has long been a subject of interest to researchers and government policymakers [4–7]. Previous studies have revealed the different health impacts of both the physical aspects of housing, such as housing condition and building design, and the socioeconomic aspects, such as tenure, affordability, and neighborhood [8–14]. Among the many potential influences on health, housing tenure is an essential factor that cannot be disregarded. Existing research generally agrees that housing tenure type plays an important role in health [15, 16]. However, this relationship between tenure type and health has been debated [17]. Some previous studies have suggested that homeowners have better health than renters [18, 19], while others have shown the opposite results [4]. On the one hand, ownership of one’s own home can bring a sense of social identity and stability, thereby reducing psychological insecurity [20]. In addition, for the children of migrants, a stable home provides a stable environment for growth and learning, which is conducive to healthy physical and mental development [21]. On the other hand, however, in the context of China’s social reality, as urbanization continues to accelerate and housing prices rise, the relatively low incomes of the migrant population and the difficulty of accessing financial support, such as mortgages or housing provident funds, can create significant pressure to purchase and own a home in the city, which may have a negative impact on the health status of the migrant population [22]. At the same time, home ownership competes with the need for food, education, and health care [23–25], placing greater employment pressures and financial burdens on migrants. Therefore, it is necessary to investigate the underlying mechanisms how housing tenure impacts health.

However, only a few studies tended to explore the pathways of the association between housing tenure and health status [14, 26, 27]. A Study in Germany found that physical and social features of home and neighborhood partially mediated the association between housing tenure and self-rated health [26]. Also, social environment, such as social capital and social control, mediated the association between housing tenure and mental health [14]. Study in Korea found that satisfaction with economic conditions mediated the association between homeownership and mental health [27].

In addition to the above mediators, health service utilization and social integration also serve as pathways through which housing tenure is associated with migrants’ health outcomes. On the one hand, previous research has indicated that improving the utilization of health services and increasing the diversity of health service providers contribute to better health outcomes [28–30]. Higher levels of social inclusion tend to be accompanied by better health outcomes [31]. On the other hand, due to the institutional discrimination (i.e., the unequal rights between homeowners and renters) embedded in China’s housing system [32], migrant renters cannot obtain equal access to basic public health services (e.g., health education, health records, and free medical check-ups) like migrant homeowners and experienced social exclusion [33, 34]. Meanwhile, homeownership has a positive impact on the quality of life, social-economic development, and stability, as well as the social harmony and stability of individuals and families, thereby playing an important role in social integration [35]. However, to our knowledge, no previous study specifically examined the mediation effects of health service utilization and social integration on the association between housing tenure and health. Given that the two pathways could inform diverse policies and interventions to safeguard and enhance migrants’ health, it is imperative to conduct evidence-based research to examine the existence and significance of the two pathways.

**Fig. 1:**
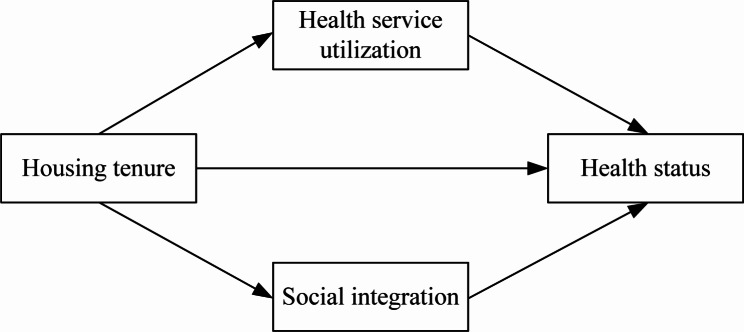
Graphic representation of the conceptual framework

This study proposes to examine the impact of the housing tenure of migrants on their health status and to explore the mediating effect of health service utilization and social integration in this context using data from the 2017 China Migrants Dynamic Survey. The conceptual framework is illustrated in Fig. 1. Specifically, two clear questions are addressed: (1) What is the impact of housing tenure on the health status of migrants? (2) Whether housing tenure indirectly affects the health status of migrants through health service utilization and social integration? This study examines the mechanisms through which housing tenure affects the health status of the migrant population in China from both an economic and a sociological perspective and provides recommendations to promote the implementation of relevant policies and measures.

## Materials and methods

### Data source and sample

**Fig. 2:**
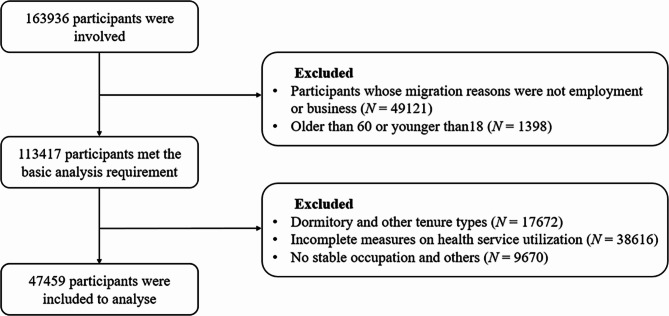
The data processing and analysis flowchart of this study

We utilized the 2017 China Migrants Dynamic Survey data, which was commissioned by the National Health Commission and coordinated by the China Population and Development Research Centre. This survey employed a stratified three-stage probability-proportionate‐to‐size (PPS) sampling method for sampling investigation. The targets of the survey were migrants who were 15 years old and over and who had resided locally for more than one month and held household registrations outside of the host city. This survey covers a total of 16,3969 migrants in all 32 provinces of China, 351 cities, and 8,500 communities or villages. The questionnaire contains basic information of the respondents and their family members, employment status, migration status, health and public services, and social integration, which is valuable for health-related research. In this study, we selected migrants 18–60 years of age. Although the survey records information about the entire household, this study only investigated the economic migration of the household. Migrants whose reason for migration is not employment or business were excluded. Observations with missing values in the variables of interest were also dropped. Finally, 47,459 observations were deemed eligible for inclusion. The flowchart of observations is shown in Fig. 2.

### Measurements

#### Self-rated health

Our health outcome was self-rated health, which was measured by the question “How is your health condition?” The responses included “healthy”, “largely healthy”, “unhealthy, but can take care of myself”, and “unable to take care of myself”. Due to the skewed distribution of responses, we treated “health” as “good” health and the rest as “poor” health. Hence, our health outcome was a dummy variable.

#### Housing tenure

Housing tenure was our primary explanatory variable and was gauged by the questions “What is the nature of your current residence?” and “What was the average monthly housing expenditure for your household in the local area over the past year?”

Consistent with prior literature [36], housing tenure was divided into three categories: private renters (renting from private housing), owners with mortgages (homeowners with mortgages), and outright owners (homeowners without mortgages).

#### Health service utilization and social integration as mediators

Combining existing literature [37, 38], health service utilization was measured by multiple dimensions, such as health education, health records, and access to public health care. These dimensions were reflected through three questions “Have you been given a local health record?”, “In the past year, have you received health education in your current village/community in the following areas?” and “How long does it take to get to the nearest public health service (including community health centers, village clinics, hospitals, etc.) from where you live?”. A standardized composite score was calculated to measure health service utilization by adding the values of these three variables after standardization.

Align with previous literature [39], social integration was measured by migrants’ perceptions of integration including economic status, social communication, acculturation, and self-identity. These dimensions were reflected through an eight-question scale, which was evaluated using a four-point Likert scale. We scored the degree of agreement produced by the participants as 1 (strongly disagree), 2 (disagree), 3 (generally agree), 4 (strongly agree), of which five items were positive and three items were negative feelings. After reversing the scores of the negative feelings items and summarizing the total items, a standardized composite score was added to measure the level of social integration, with the higher value reflecting a higher level of social integration.

#### Covariates

With reference to previous studies on housing and health [14, 16], we employed sociodemographic and socioeconomic factors as covariates that may influence health outcomes. Sociodemographic factors included gender, marital status, and age. Socioeconomic factors included education, occupation, and household income (monthly income scaled by ¥1000). Specifically, gender was coded as 1 if the participant is male and 0 otherwise. Marital status was assigned a value of 1 if married and 0 otherwise. Age was used as a continuous variable. Education was divided into three levels from low to high: junior high school or lower, senior high school, and college or higher. According to the occupational division method widely used in China [40–42], migrants’ occupations were divided into four categories: manual workers, service personnel, self-employed workers, and professionals.

### Statistical analysis

To examine the total, direct, and indirect effects of housing tenure on health, we conducted the Causal Steps Approach [43]. First, we performed a binary logistic regression model to examine the total effect of housing tenure on health. Second, we added health service utilization and social integration in the binary logistic regression model to examine the direct effects of housing tenure, health service utilization, and social integration on health. Third, we further conducted two ordinary least squares regression models with health service utilization and social integration as dependent variables to examine whether housing tenure affected health service utilization and social integration. We controlled sociodemographic and socioeconomic factors in all the models.

This study used the bootstrap method to test the mediating effect of health service utilization and social integration. Since this study tested two mediator variables, to explore which mediator variable contributes the most to the indirect effect, we also used the KHB method [44]. For linear models, unstandardized coefficients (*β*) were reported, and for logistic models, odds ratios (OR) were reported. Stata/MP 17.0 was used to perform all data analyzes with a two-tailed *p* value < 0.05 as the level of significance.

## Results

### Descriptive statistical

Table 1 presents the descriptive statistics of the variables used in the analysis. In this sample, 85.58% of the migrants reported themselves as having good self-rated health. Moreover, the housing tenure is mainly private renter, accounting for 73.78%. After standardization, the mean scores for health service utilization and social integration for migrants were 0.565 ± 0.241 and 0.708 ± 0.138, respectively.

**Table 1:** Baseline information of the migrants, China, 2017 (*N* = 47,459)

Variables	M/%	SD	Min	Max
Self-rated health				
Poor	14.42%	-		
Good	85.58%	-		
Housing tenure				
Private renters	73.78%	-		
Owners with mortgages	14.51%	-		
Outright owners	11.71%	-		
Social integration	0.708	0.138	0	1
Health service utilization	0.565	0.241	0	1
Age	35.433	8.897	18	60
Gender				
Female	40.96%	-		
Male	59.04%	-		
Marital status				
Single	18.42%	-		
Married	81.58%	-		
Education				
Junior high school or lower	54.56%	-		
Senior high school	21.88%	-		
College or higher	23.57%	-		
Occupation				
Manual workers	29.47%	-		
Service personnel	42.58%	-		
Self-employed workers	11.39%	-		
Professionals	16.55%	-		
Household income (thousand yuan)	7.679	5.735	0.5	180

### The impact of housing tenure, health service utilization and social integration on the health status of migrant populations

The association between housing tenure and health status, health service utilization, and social integration are illustrated in detail in Table 2. Model 1 shows the total effect of housing tenure on health. Compared to private renters, owners with mortgages (OR: 0.828, 95% CI: 0.765–0.896) were significantly associated with a higher risk of poor health. Compared with private renters, outright owners were associated with a lower risk of poor health (OR: 1.016, 95% CI: 0.935, 1.104), but the association was not statistically significant (*p* > 0.05). Model 2 illustrates the direct effects of housing tenure, health service, and social integration on health. Compared to private renters, owners with mortgages (OR: 0.751, 95% CI: 0.693, 0.813) was still associated with a higher risk of poor health. Compared with private renters, outright owners were associated with a higher risk of poor health (OR: 0.903, 95% CI: 0.829, 0.983). Moreover, health service utilization (OR: 1.422, 95% CI: 1.268, 1.594) and social integration (OR: 4.357, 95% CI: 3.555, 5.341) were both significantly associated with a higher probability of good health. Model 3 and Model 4 demonstrate that the type of housing tenure was significantly associated with health service utilization and social integration. Specifically, owners with mortgages and outright owners were positively associated with health service utilization (owners with mortgages: *β* = 0.032, 95% CI: 0.026, 0.039; outright owners: *β* = 0.041, 95% CI: 0.034, 0.048) and social integration (owners with mortgages: *β* = 0.054, 95% CI: 0.051, 0.058; outright owners: *β* = 0.068, 95% CI: 0.064, 0.072) compared to private renters.

**Table 2:** The associations of housing tenure with health outcome, health service utilization and social integration, with “poor” health as references, among migrants, China, 2017 (*N* = 47,459)

	Model 1	Model 2	Model 3	Model 4
	Self-rated health	Self-rated health	Health service utilization	Social integration
	OR (95% CI)	OR (95% CI)	*β* (95% CI)	*β* (95% CI)
Housing tenure (ref: private renters)				
Owners with mortgages	0.828^***^	0.751^***^	0.032^***^	0.054^***^
	(0.765, 0.896)	(0.693, 0.813)	(0.026, 0.039)	(0.051, 0.058)
Outright owners	1.016	0.903^*^	0.041^***^	0.068^***^
	(0.935, 1.104)	(0.829, 0.983)	(0.034, 0.048)	(0.064, 0.072)
Health service utilization	-	1.422^***^	-	-
	-	(1.268, 1.594)	-	-
Social integration	-	4.357^***^	-	-
	-	(3.555, 5.341)	-	-
Age	0.948^***^	0.947^***^	-0.001^***^	0.001^***^
	(0.945, 0.951)	(0.944, 0.950)	(0.001, 0.001)	(0.001, 0.001)
Gender (ref: female)	1.162^***^	1.158^***^	-0.008^***^	0.003^*^
	(1.100, 1.226)	(1.096, 1.222)	(-0.013, -0.004)	(0.001, 0.005)
Marital (ref: single)	0.960	0.951	0.044^***^	-0.003^*^
Married	(0.883, 1.042)	(0.875, 1.034)	(0.038, 0.050)	(-0.007, -0.000)
Education (ref: junior high school or lower)				
Senior high school	1.094^*^	1.043	0.039^***^	0.023^***^
	(1.020, 1.174)	(0.972, 1.119)	(0.033, 0.045)	(0.020, 0.026)
College or higher	1.166^***^	1.075	0.046^***^	0.045^***^
	(1.070, 1.272)	(0.986, 1.174)	(0.039, 0.052)	(0.042, 0.049)
Occupation (ref: manual workers)				
service personnel	0.996	0.959	0.009^**^	0.024^***^
	(0.936, 1.060)	(0.900, 1.021)	(0.004, 0.015)	(0.021, 0.027)
self-employed workers	1.061	1.030	0.004	0.020^***^
	(0.970, 1.160)	(0.941, 1.126)	(-0.004, 0.012)	(0.015, 0.024)
professionals	1.092	1.050	0.026^***^	0.022^***^
	(0.992, 1.202)	(0.954, 1.156)	(0.019, 0.033)	(0.018, 0.026)
Household income	1.014^***^	1.015^***^	-0.004^***^	0.000^**^
	(1.008, 1.020)	(1.009, 1.021)	(-0.004, -0.003)	(0.000, 0.001)

Unstandardized *β* coefficients and odds ratios (OR) and 95% confidence intervals (95% CI) were reported. ^*^p < 0.05, ^**^p < 0.01, ^***^p < 0.001.

### Mediating effects of housing tenure on health status through health service utilization and social integration

It can be seen from Table 3 that the confidence intervals for the indirect effects of health service utilization and social integration did not include zero, and there were mediating effects. Table 3 also shows the separate indirect effect size for each mediating variable, as well as in the full model. Adding health service utilization to the mediation model alone, the indirect effects (owners with mortgages: *β* = 0.016; outright owners: *β* = 0.020) were significant (*p* < 0.001). Adding social integration to the mediation model alone, the indirect effects (owners with mortgages: *β* = 0.086; outright owners: *β* = 0.108) were significant (*p* < 0.001). When adding both factors into the mediation model, the indirect effects (owners with mortgages: *β* = 0.091; outright owners: *β* = 0.115) of housing tenure were significant (*p* < 0.001). Between the mediators, social integration contributes more to the indirect effect than health service utilization.

**Table 3:** The mediating effects of health service utilization and social integration, among migrants, China, 2017 (*N* = 47,459)

Housing tenure (ref: private renters)	Mediation effects based on Bootstrap method		Mediation effects based on KHB method		
	health service utilization	social integration	health service utilization	social integration	health service utilization & social integration
	*β* (95% CI^a^)	*β* (95% CI^a^)	*β* (95% CI)	*β* (95% CI)	*β* (95% CI)
Owners with mortgages	0.016^***^	0.086^***^	0.016^***^	0.086^***^	0.091^***^
	(0.011, 0.021)	(0.074, 0.098)	(0.010, 0.022)	(0.072, 0.100)	(0.077, 0.105)
Outright owners	0.020^***^	0.108^***^	0.020^***^	0.108^***^	0.115^***^
	(0.015, 0.026)	(0.093, 0.123)	(0.014, 0.027)	(0.092, 0.123)	(0.098, 0.131)

Sociodemographic factors and socioeconomic factors were adjusted. CI^a^: Confidence interval with 5000 bootstrap samples. ^*^p < 0.05, ^**^p < 0.01, ^***^p < 0.001

## Discussion

This study examined the effect of the type of housing tenure on migrants’ health and the pathways of the effect. We found important disparities in health among participants with different types of housing tenure, even after taking into account sociodemographic and socioeconomic factors. Specifically, owners with mortgages had a lower probability of good health than private renters, while there is no significant difference in health between outright owners and private renters. Additionally, we found that the health effect of housing tenure was partially mediated by migrants’ health service utilization and social integration.

There may be several potential explanations for the different health effects of housing tenure. When migrants plan to purchase their own housing, they often have to work longer hours to increase their income, while long working hours have a negative impact on health [45]. Moreover, buying a home creates a lot of psychological stress for migrants, which also impedes their health [46]. These may explain why owners with mortgages had a lower probability of good health than private renters. In addition, when migrant homeowners pay off their loan and become outright owners, they face a smaller risk of housing insecurity and do not experience housing affordability stress than private renters, which could lead to better health outcomes [47–50]. Homeownership, in itself, can be beneficial for increasing psychological benefits such as emotional stability and a sense of control, which is beneficial for health status [51, 52]. Yet, saving for home ownership competes with other expenses for health-related necessities such as healthy food and healthcare, which causes damage to health [4]. These may explain why there is no significant difference in health between outright owners and private renters.

We extended previous studies by examining the mediation effect of health service utilization and social integration on the association between housing tenure and migrants’ health. We found that homeownership was positively associated with health service utilization, which improved migrants’ health. This may be because, due to the unequal rights between homeowners and renters, migrants who own a home in the host city are positively related to the establishment of a health record and participation in health education, leading to a higher level of health service utilization than migrant renters [33].

Good health service utilization also further promotes migrants’ health. First, good health service utilization is often accompanied by improved disease prevention, better health literacy, higher health care spending, and more convenient health care services, all of which can contribute to better health. A previous study found that states with a higher ratio of social to health spending had significantly better subsequent health outcomes [53]. Second, good utilization of health services can provide better treatment and rehabilitation services, such as surgery and physiotherapy, which likely reduces the adverse health impact of disease. A study in Northwest Ethiopia found that maternal health services significantly reduced mortality rates among pregnant women [54].

Moreover, we found that owners with and without a mortgage were positively associated with a higher social integration level than private renters, which led to a higher probability of good health. First, the higher likelihood of a high social integration level may be because homeownership can be considered an emotional investment and indicates a sense of membership in the host city [55], which could improve migrants’ sense of belonging and make them more easily involved in communities. Second, homeownership can also be considered a financial investment [56]. When housing prices rise rapidly, owning a home represents an increase in household wealth [57], which could enhance the sense of control and self-identity of migrants. Third, from the perspective of residence‑based housing [6], owning a home in the host city means residential stability and can promote a sense of security and settlement intention. All of these can facilitate migrants’ social integration.

A high social integration level further promotes migrants’ health. This may be because a high level of social integration leads to a healthier psychological state, a better social mentality, and less perceived stress, which increases the probability of good health. For example, a previous study found that a high level of social integration increased the sense of personal control, belonging, and trust [31]. In addition, a high social integration level means improved social networks, increased personal identity, and greater community support, all of which may be linked with better health status [58].

As a fundamental guarantee for human survival, housing is especially important for China’s massive population of migrant workers, who constitute one of the country’s largest labor forces. Therefore, the relationship between housing and migrant populations is crucial for China’s development. The different effects of housing tenure on health provide more direct insights. The government should accelerate the reform of housing policy, gradually narrowing the gap between homeowners and renters [59]. For instance, it should actively implement equal rights for rent and purchase to alleviate inequality, promote the construction of indemnificatory housing to improve housing conditions, and provide housing subsidies to improve housing payment capacity for those in housing difficulties. Our mediation analysis results suggested that good health service utilization and a high social integration level were important for migrants’ health promotion. Hence, to better satisfy migrants’ health service needs and reduce the health disparities between renters and homeowners, the government could ask communities to develop a more comprehensive health service utilization system based on social policy development. For social integration, promotions or incentives should be conducted for migrants to participate in these local social activities, such as community sports, to boost their self-identity and sense of belonging [60, 61].

A major strength of this study is that we included a large sample of migrant populations across China, which enhances the persuasiveness of the research findings. Additionally, compared to other research on the mediating effects of housing tenure on health, we have found that health service utilization and social integration were two appropriate mediating variables, which have important sociological implications. This study also has some limitations. Firstly, the use of cross-sectional data in this study was insufficient to establish a causal relationship between housing tenure and health. Secondly, although confounding factors were adjusted for in the model, the absence of information on psychological (e.g., childhood abuse, trauma, or neglect), health behavior (e.g., smoking, drinking, and exercising), and environmental (e.g., neighborhood relations, community health, and housing characteristics) factors may still lead to potential confounding. Thirdly, the measurement of health services was imperfect due to limitations in the available data. Fourthly, while the questionnaire exhibited good reliability and validity, subjective issues could not be completely eliminated. As mentioned earlier, the factors that influence health are complex, and even though mediation analysis was employed to provide a more logical approach to analysis, it may not be sufficiently comprehensive. Follow-up studies are needed in the future to address these gaps.

## Conclusions

In conclusion, among the migrant populations, homeowners with mortgages had a lower likelihood of good health than private renters, while there was no significant difference in the health status between outright owners and private renters. Moreover, health service utilization and social integration mediate the effects of housing tenure on the health status of migrant populations. Better health service utilization and higher levels of social integration are associated with a higher probability of being healthy. Policies and interventions can be designed to improve the health service utilization and social integration of migrant populations and reduce health disparities across different housing tenure types.

## Data Availability

The datasets analyzed during the current study are available in the China Migrants Dynamic Survey (2017), which was commissioned by the National Health Commission and coordinated by the China Population and Development Research Centre. https://chinaldrk.org.cn/wjw/#/home.

## Abbreviations

OR: Odds Ratio
CI: Confidence interval
CI^a^: Confidence interval with 5000 bootstrap samples
